# Three-dimensional foot trajectory in female patients with end-stage hip osteoarthritis during walking

**DOI:** 10.1038/s41598-022-14070-8

**Published:** 2022-06-14

**Authors:** Yu Kiko, Taiki Ogata, Hirotaka Uchitomi, Masaaki Matsubara, Yoshihiro Miyake, Yoshiaki Wada

**Affiliations:** 1 Department of Rehabilitation, Nissan Tamagawa Hospital, Tokyo, 158-0095 Japan; 2 Department of Computer Science, Tokyo Institute of Technology, Kanagawa, 226-8503 Japan; 3 Department of Orthopedic Surgery, Nissan Tamagawa Hospital, Tokyo, 158-0095 Japan

**Keywords:** Diseases, Health care

## Abstract

Osteoarthritis (OA) is a risk factor for falls. To decrease the fall risk, it is important to evaluate the detailed features of the gait of patients with OA. This study aimed to investigate the spatio-temporal parameters of gait in patients with end-stage hip OA, especially foot trajectory. We measured normal speed gait in patients with hip OA and in healthy controls (HCs) using inertial measurement units attached to shanks. The stride lengths in the affected and unaffected sides in the patients with hip OA were shorter than those in the HCs, but the position of maximum foot clearance was not significantly different between the two groups. The patients with hip OA compensated the position of maximum foot clearance to avoid fall risk. The horizontal plane foot trajectory in patients with hip OA suggests that the lateral bending of the trunk during walking, which is frequently seen in them, was a result of the lateral distance at swing down being located significantly more medially on the unaffected side than on the affected side. Herein, a new gait parameter of lateral distance at swing was discovered by a detailed evaluation of three-dimensional foot trajectory of female patients with end-stage hip OA.

## Introduction

Hip osteoarthritis(OA) is common in females^1^ and is a chronic progressive disease characterised by the degeneration and wear of articular cartilage, resulting in joint destruction and reactive bone growth (osteochondria and osteophytes). Limited range of motion (ROM), inguinal pain and limping are characteristic^2–5^, and their effects cause activity limitation^6^. The patients with hip OA have a higher risk of death than healthy individuals, and the history of diabetes, cancer, or cardiovascular disease and gait disorders are listed as major risk factors^7^. In addition, OA is also known to be a risk factor for falls^8^. However, it is not well understood what kind of walking problem the OA patients have. The association between fall risk and pain and dysfunction has been modest and not associated with radiographic OA^9^. In addition, female patients with end-stage hip OA have a higher incidence of falls within 1 year, and an association between fall risk and limping and knee extensor strength has been reported, but detailed gait evaluation has not been performed^10^. To decrease the fall risk during walking, it is important to understand how hip OA affects gait features in detail. In addition, recently, it has been reported that abnormal hip joint loading is involved in the progression of hip OA^11^. The hip joint loading is potentially modifiable with interventions using gait training^12,13^, which requires appropriate gait instruction and adjustment of gait volume. For the appropriate instruction, it is also important to evaluate the gait of the patients with hip OA accurately and in detail.

Regarding gait characteristics of patients with hip OA, it is well-known that walking speed decreases due to short stride length and short affected limb step^14–17^. When walking on the treadmill at constant speed, the step length on the affected side is longer than that on the unaffected side.^18–20^. Many researchers reports on the walking speed of patients with hip OA^14–17,21,22^; however, reports comparing the stride speed of the affected and unaffected limbs are rare. There are various phases in the gait cycle such as swing and stance; the gait is generated by the rhythmic combination of these phases. Patients with hip OA have an asymmetry in step and swing duration between affected and unaffected limbs caused by short step^19,21,22^ and stance durations^18,23^ in the affected limb. The increase in step length asymmetry is associated with a decrease in mechanical energy exchange^20^. In addition, the external hip flexion, extension, and adduction moments during walking are lower in patietns with hip OA^24^, and extension angles of the hip and knee in the latter half of the stance phase are smaller than those in healthy controls(HCs), but there is no difference in the ankle joint angle^25^. The peaks of hip flexion and extension angles decrease in the affected limb relative to those in the unaffected limb^26^. In patients with hip OA, the variability of walking on the treadmill was higher on the affected side than in the HCs, but the variability was lowest when the gait speed was close to the self-selected walking speed^27^. The variability of stride duration decreased significantly 1 year after total hip arthroplasty (THA) compared with that preoperatively and was comparable to that of the HCs^28^.

As seen in these previous studies, various gait characteristics of hip OA have been reported. However, to the best of our knowledge, there is no report that investigates the gait characteristics of patients with hip OA in detail using three-dimensional foot trajectory. Since the gait parameters of patients with hip OA are asymmetric the foot trajectory is likely also asymmetric. The three-dimensional foot trajectory shows features in the sagittal, frontal, and horizontal planes, but gait features in the horizontal plane were not almost reported. Patients with hip OA frequently show an exaggerated lateral bending of the trunk during gait. The lateral bending of the trunk may be characteristic of the foot trajectory, especially during the swing phase.

This study aimed to evaluate the three-dimensional foot trajectory of female patients with end-stage hip OA on the affected and unaffected limbs and to clarify the gait characteristics in comparison with HC. The average prevalence of hip OA in Japan is 1.0%, but is higher in females (2.0%) than males (0%)^1^. Male patients were excluded in this study to avoid possible confounding due to unbalanced sex distribution. The evaluation of three-dimensional foot trajectory provides multiple gait features such as the sagittal, horizontal, and frontal planes of the spatio-temporal parameters. In recent years, there has been an increase in gait analysis with wearable inertial measurement unit (IMU) sensors that have provided an accessible and affordable alternative to traditional optical gait analysis systems^29^. Mao et al. proposed a system to estimate the foot trajectory in three dimensions during walking by attaching the IMUs on shanks just above both ankles^30^. The gait indices such as the stride length, the position of maximum foot clearance (maximum foot clearance), and speed estimated by this system strongly correlate with those measured by the optical motion-capture system. This system can also evaluate stride duration, stance duration and swing duration for the right and left lower limbs. In this study, we use this wearable system to evaluate the gait characteristics of patients with hip OA.

## Methods

### Participants

The participants were 50 outpatients with unilateral end-stage hip OA at the Department of Orthopedic Surgery, Nissan Koseikai Tamagawa Hospital, (25 LOA ; 25 ROA ; all females) in 2018-2019. We calculated that with a sample of 75 participants, the study would have 80% power to detect a 0.17 effect size, with a type I error rate of 5%. The patients had a minimal joint space (MJS) of less than 2 mm and a low Japan Orthopaedic Association (JOA) hip score, and were considered severe enough to require THA. We defined these patients as end-stage. The HC consisted of 25 age-matched females who were independent in their daily activities and able to walk on their own. We excluded the patients if they had neurological, vascular, and other lower extremity musculoskeletal conditions or psychiatric disorders that affected gait or functional performance. We also excluded patients with self-reported lack of sensation in the foot or lower limb and those who were unable to walk short distances (30 m) without an assistive device and patients with pain in the hip on the unaffected side. This study was approved by the Institutional Review Board of Nissan Tamagawa Hospital and the Tokyo Institute of Technology for research ethics committee. All participants provided written informed consent before participation. All methods were performed in accordance with the Helsinki Declaration and relevant guidelines and regulations. There were no differences in age, height, weight, or BMI between patients with hip OA and HCs (Table 1). The hip flexion, extension, abduction, adduction, external rotation, and internal rotation ROM angles, hip abduction muscle strength, knee extension muscle strength, MJS, and JOA Hip score were significantly different between the affected and unaffected sides for the patients with hip OA (Supplementary Table S3). There was no significant difference of affected sides between the patients with LOA and ROA except for hip abduction (Supplementary Table S4). In addition, there was no significant difference in unaffected sides between the patients with LOA and ROA. There was no significant difference in the stage of hip OA between the affected and unaffected sides of patients with LOA and ROA (Supplementary Table S5).

**Table 1:** Participant demographics.

Variables	Mean (SD)			p-value
	HC (N = 25)	LOA (N = 25)	ROA (N = 25)	
Age (years)	60.9 (7.0)	63.4 (7.3)	65.5 (7.6)	0.090
Height (cm)	155.7 (6.2)	154.4 (4.5)	154.3 (6.5)	0.618
Weight (kg)	53.3 (8.9)	53.9 (8.4)	54.2 (8.9)	0.865
BMI	22.0 (3.3)	22.6 (3.2)	22.6 (2.5)	0.544

The means and standard deviations of age, height, weight and BMI for each group.

The hip joint ROM angles were determined by passively moving the patients’ legs and measuring the maximum angles using a goniometer. The pelvis was stabilised to prevent rotation or tilting^31^ when passive ROM was measured by an experienced physical therapist. The measuring was conducted with the patients in the supine position except for measuring the external and internal rotation angles. The external and internal rotation angles were measured in the prone position at 90$^\circ$ flexion knee joint. The hip abductor strength was measured using a hand-held dynamometer ($\mu$TAS F-1, Anima Co., Ltd.) in the supine position. The sensor pad was fixed to the distal thigh. The maximum isometric contraction for 5 seconds was performed three times on each side, and the maximum value was determined as the weight ratio (kgf/kg). The knee extension strength was also measured using the hand-held dynamometer. The sensor pad was fixed at the distal leg in a seated lower leg droop position, and the maximum isometric contraction for 5 seconds was performed three times on each side. The maximum value was measured as the ratio of body weight (kgf/kg). MJS was measured by an orthopaedic surgeon using a simple radiograph. In addition, the severity of the patients with hip OA were measured by the JOA hip score, a physician-completed disease-specific scale consisting of four items: pain (40 points), ROM (20 points), ability to walk (20 points), and activities of daily living (20 points). The JOA hip score was used because the JOA score is designed in term of the Japanese life style. In addition, it was reported that there was no significant difference in total score between the Harris hip score and the JOA hip score, and there was a strong correlation between them^32^. Hip pain during walking was measured using the visual analogue scale. The stage of hip OA was classified by orthopaedic surgeons based on the Japanese Orthopedic Association’s osteoarthritis staging system.

### Apparatus and gait measurement

**Figure 1:**
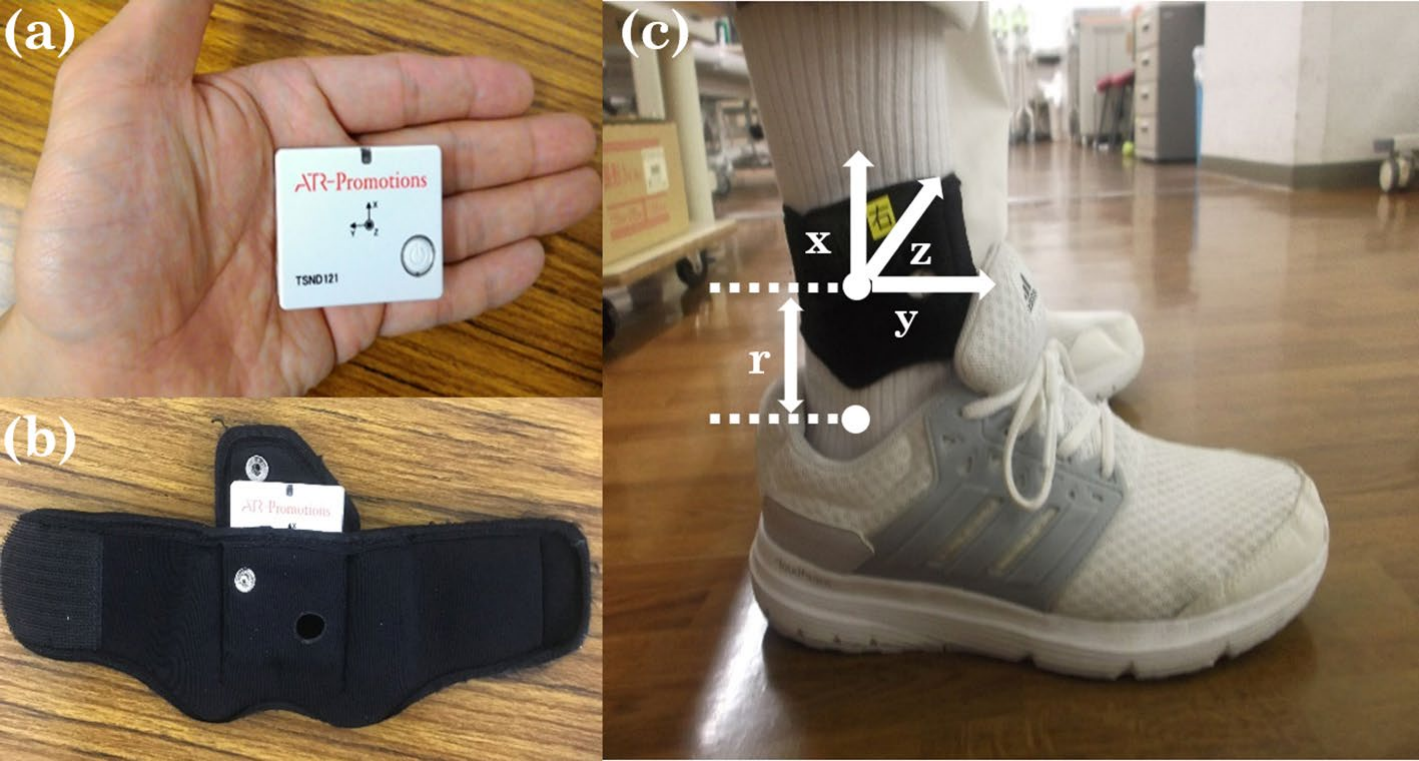
Configuration of the IMUs. (**a**) The IMU with the accelerometer and gyroscope. (**b**) The special band for wearing the IMU on the shank. (**c**) The position and coordination of the IMU. Two IMUs are attached to the shank just above the malleolus at a distance of r. IMUs are attached to the shank in the position 0.03 m above the malleolus. The axes x, y, and z are the coordinate system of the IMU, where the z-axis is perpendicular to the sagittal plane formed by the y and z-axes.

The IMUs (TSND121, ATR-Promotions) that were used for measured gait are shown in Fig. 1a. Using this IMU, we measured the acceleration and angular velocity to estimate the gait parameters. The two IMUs were attached on the shanks on both sides just above the malleolus using a special band (Fig. 1b). The attachment position and coordinate system of the IMUs are shown in Fig. 1c. The x , y , and z axes represented inferior/superior, posterior/anterior, and medial/lateral directions, respectively. The measurement ranges of acceleration and angular velocity were ±8 G and ±1000 dps, respectively, and the sampling frequency was set to 100 Hz. The size of the TSND121 is 37 mm $\times$ 46 mm $\times$ 12 mm and the weight is approximately 22 g.

We estimated spatio-temporal gait parameters using the method proposed by Mao et al^30^. Spatio-temporal parameters were estimated via the double integration of the linear acceleration transformed by the IMU orientation information. To reduce the integral drift error, an inverted pendulum model, applied with a linear error model, was introduced at the mid-stance to estimate the update velocity^30^. The motion of the IMU at the mid-stance can be modelled as a rotational motion in a three-dimensional space. The position vector r and angular velocity in the laboratory coordinate frame were computed from the estimated orientation of IMU^30^. By this method, we estimated the maximum foot clearance, stride length, stride speed, stride duration, stance duration and swing duration for left and right lower limbs.

### Task and procedure

For the measurements, participants walked about 30 m down a straight and flat corridor at their self-selected speed. The measurement was carried out with participants familiar shoes with flat soles. The number of left and right strides were measured; at least 30 for each stride. All measurements were taken once for each participant. All participants walked without any assistive devices.

### Statistical analysis

We used the fourth to the 13th stride of each leg to calculate means for each gait parameter. We aggregated each parameter for the left and right legs instead of affected and unaffected sides to simply compare the results of the LOA and ROA patients with the HCs. The mean of each spatio-temporal parameter and lateral distance at swing phase were analysed using two-way mixed ANOVA. The factors for the ANOVA were three groups (the LOA patients, ROA patients, and HCs) and two legs (left and right legs). For multiple comparisons, we used Shaffer’s method. We used the Student’s T-test and the Mann-Whitney U test for the participant demographics, affected/unaffected physical functions and JOA hip score with and without normal distribution, respectively. In addition, the stage of hip OA was analysed using the chi-square test. All statistical analyses were performed using R with the significance level set at *p* $<0.05$.

## Results

**Figure 2:**
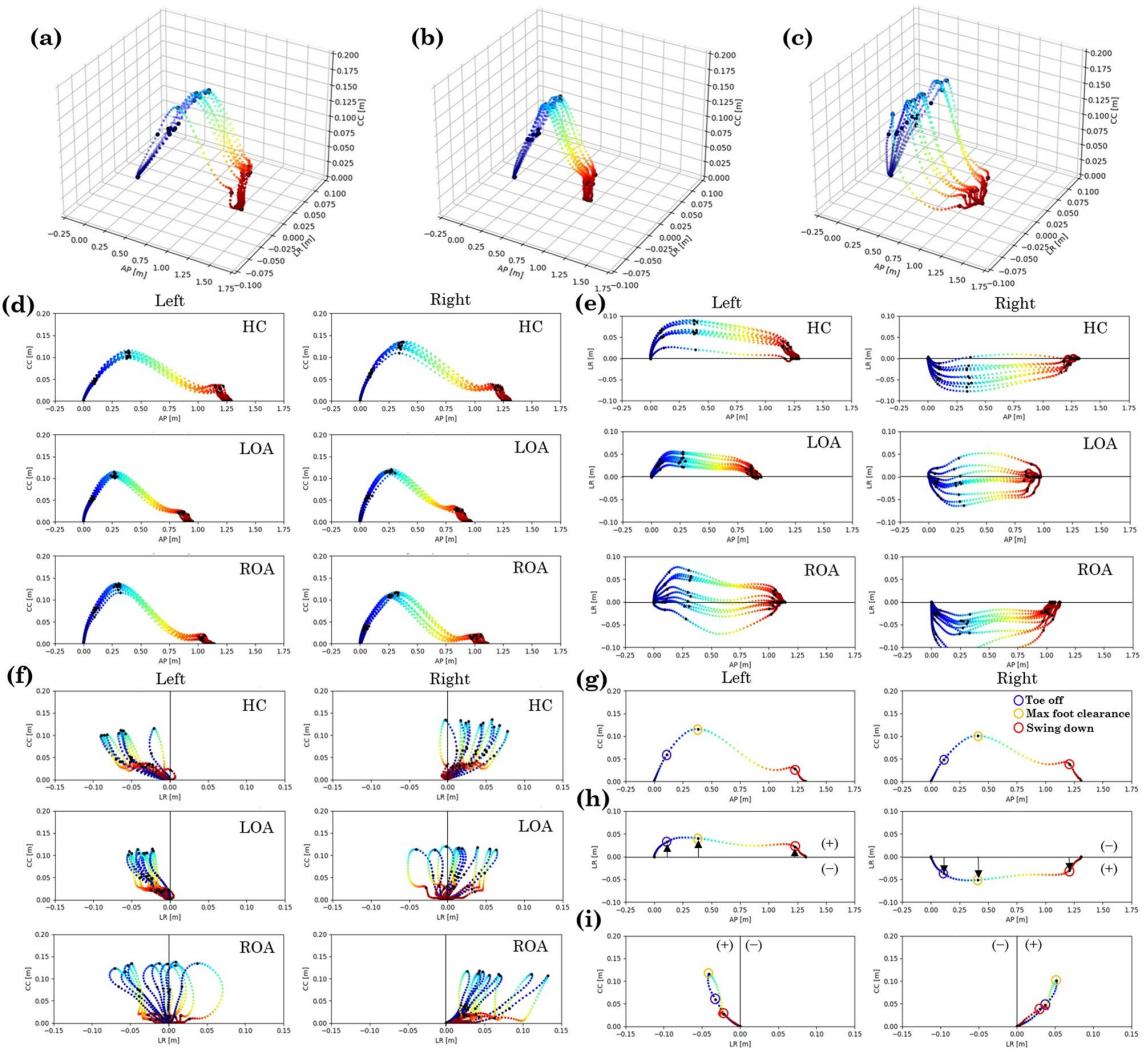
An example of the three-dimensions estimated foot trajectory obtained from shank during walking. (**a**) Three-dimensional foot trajectory of healthy control (HC) left leg, (**b**) left hip osteoarthritis (LOA) left leg, (**c**) right hip osteoarthritis (ROA) left leg. An example of (**d**) sagittal plane, (**e**) horizontal, and (**f**) coronal plane of the three-dimensional estimated foot trajectory obtained from shank during gait. An example of foot trajectory in the sagittal (**g**), horizontal (**h**), and frontal planes (**i**) for the left and right legs of HC. The black dots indicate the positions of toe off, maximum foot clearance, and swing down during the swing phase of walking (**g**–**i**). In the horizontal and frontal planes, the midline was set as 0, and the lateral distance (LD) from the midline was examined with the swing leg side as ($+$) and the stance leg side as (−) (**h**,**i**). The LD farthest from the midline to the swing legs side was defined as the maximum, and the LD closest to the stance legs side was defined as the minimum (**h**). From left to right and top to bottom: HC, LOA, ROA. Anterior-Posterior: AP, Cranio-caudal: CC, Left-right: LR.

### Foot trajectory

An example of each group in the three-dimensional foot trajectory of the left leg during walking is shown in Fig. 2a–c. An example of each group of the three-dimensional estimated foot trajectory in the sagittal, horizontal, and frontal planes during walking are shown in Fig. 2d–f. An example of foot trajectory in the sagittal (Fig. 2g), horizontal (Fig. 2h), and frontal planes (Fig. 2i) for the left and right legs of HC. The black dots indicate the positions of toe off, maximum foot clearance, and swing down during the swing phase of walking (Fig. 2g–i). In the horizontal and frontal planes, the midline was set as 0, and the lateral distance (LD) from the midline was examined with the swing leg side as (+) and the stance leg side as (-) (Fig. 2h,i). The LD farthest from the midline to the swing legs side was defined as the maximum, and the LD closest to the stance legs side was defined as the minimum (Fig. 2h). In the sagittal plane, the stride length of the patient with hip OA is shorter than that of the HC, but the maximum foot clearance does not seem to be significantly different (Fig. 2d). Therefore, the foot trajectory of the patient with hip OA shrinks only in the direction of walking. The foot trajectory in the horizontal and frontal planes of the HC and the patient with hip OA appears to be very different (Fig. 2e,f). The foot trajectory in the horizontal and frontal planes are in approximate symmetry in the HC, but in asymmetry and varied in the patient with hip OA, and the foot trajectory of the unaffected side is more medial than that of the affected side(Fig. 2e,f). In addition, the foot trajectory of the affected side of the patients of hip OA did not go medially, but spread laterally in some patients. However, the variation could be caused by individual differences(Fig. 2e,f). Therefore, the three-dimensional foot trajectory also appears to have a larger variation for the unaffected side limb of the ROA than the affected side limb of the LOA(Fig. 2b,c).

Due to space limitation, we show only important statistical results. All results of the statistical test are shown in the supplementary information.

#### Spatial gait parameters

**Figure 3:**
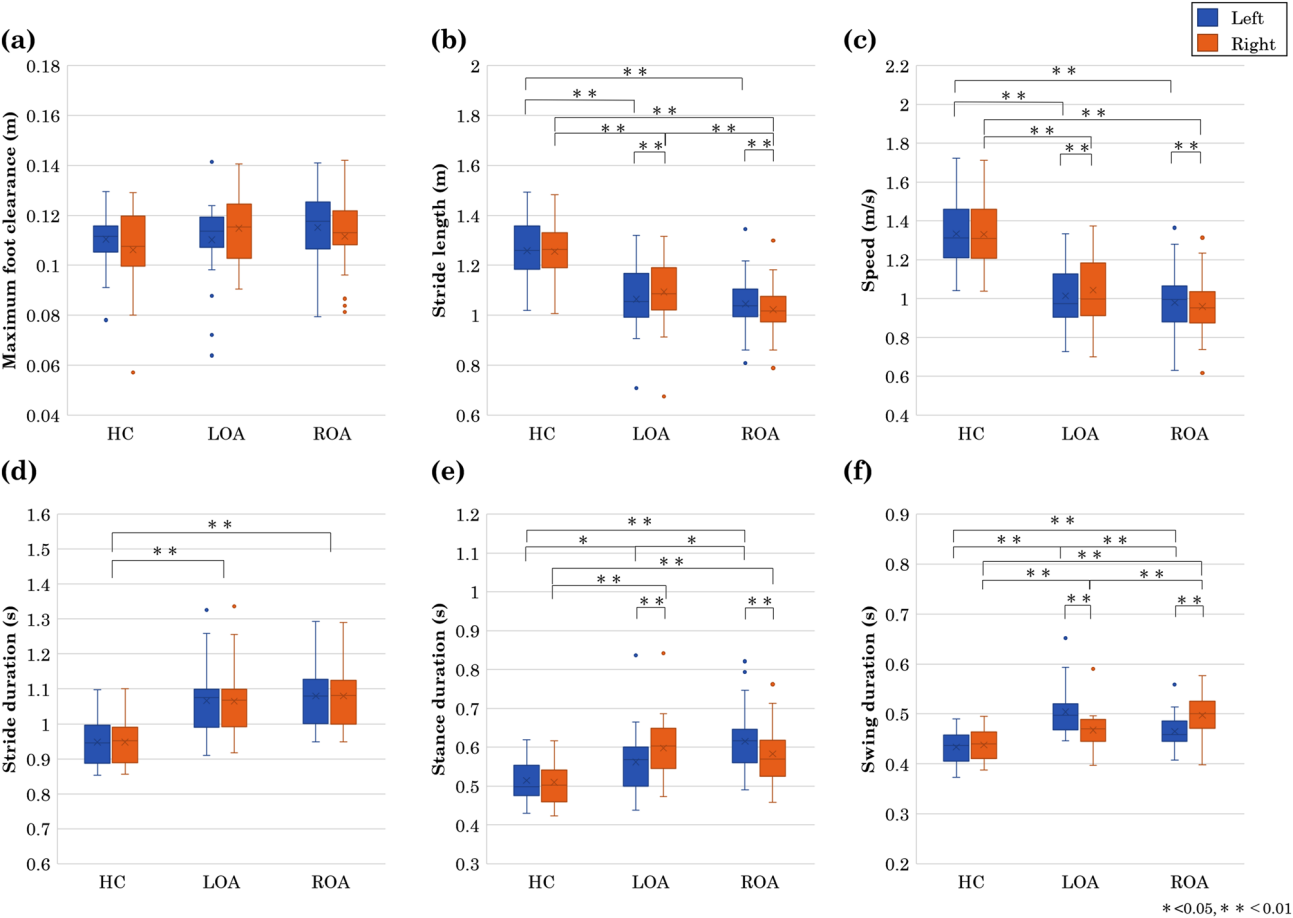
All gait parameters of (**a**) maximum foot clearance, (**b**) stride length, (**c**) speed, (**d**) stride duration, (**e**) stance duration, (**f**) swing duration. Blue is the left leg, orange is the right leg, and error bars indicate the SD. From left to right: healthy control (HC), left hip osteoarthritis (LOA), right hip osteoarthritis (ROA). * indicates significance at *p* $<\,0.05$ and ** indicates significance at *p* $<\,0.01$.

The spatial gait parameters of the left and right lower limbs for HC, LOA, and ROA, i.e., maximum foot clearance and stride length are shown in Fig. 3a,b. Mixed ANOVA was performed for the mean of each gait parameter of HC, LOA, and ROA (Supplementary Table S1). Because significant interactions were found in the maximum foot clearance ($F(2,72)=3.94$, $p=0.024$, ${\eta ^2_p}=0.099$)and stride length ($F(2,72)=11.82$, $p<0.001$, ${\eta ^2_p}=0.247$), we conducted the *post hoc* test for these gait parameters. When the simple effect of the group was found, we conducted a multiple comparison using Shaffer’s method. The results are shown in Table 2. For maximum foot clearance (Fig. 3a), there was no significant different between HC, LOA, and ROA. The stride lengths (Fig. 3b) in patients with LOA and ROA were significantly shorter in both the affected and unaffected sides than those in the HC. The stride length was significantly shorter in the affected side than in the unaffected side for both the patients with LOA and ROA.

**Figure 4:**
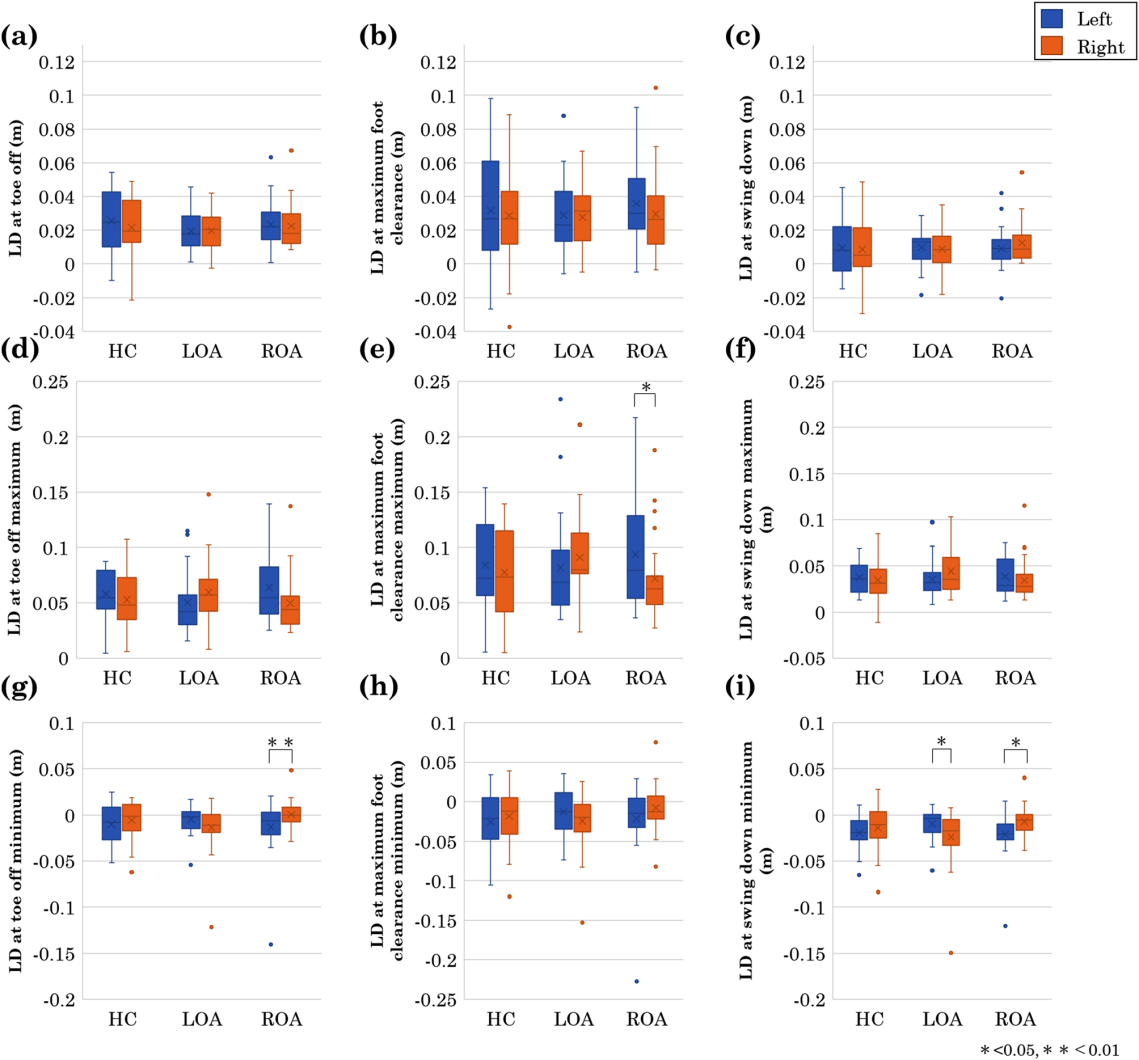
All swing phase of (**a**) lateral distance (LD) at toe off, (**b**) LD at maximum foot clearance, (**c**) LD at swing down, (**d**) LD at toe off maximum, (**e**) LD at maximum foot clearance maximum, (**f**) LD at swing down maximum, (**g**) LD at toe off minimum, (**h**) LD at maximum foot clearance minimum, (**i**) LD at swing down minimum. Blue is the left leg, orange is the right leg, and error bars indicate the participant’s SD. From left to right: healthy control (HC), left hip osteoarthritis (LOA), right hip osteoarthritis (ROA). * indicates significance at *p* $<\,0.05$ and ** indicates significance at *p* $<\,0.01$.

For the LD at swing phase of spatial gait parameters, the mean, maximum and minimum LDs of the left and right lower limbs of the HC, and the patients with LOA and ROA from the horizontal plane during toe off, maximum foot clearance, and swing down are shown in Fig. 4. Mixed ANOVA was also performed for the mean, maximum and minimum of each swing phase of LDs of the HC and the patients with LOA and ROA (Supplementary Table S2). Because significant interactions were found in the LD at toe off maximum ($F(2,72)=3.70$, $p=0.029$, ${\eta ^2_p}=0.093$), toe off minimum ($F(2,72)=3.59$, $p=0.033$, ${\eta ^2_p}=0.091$), and swing down minimum ($F(2,72)=7.20$, $p=0.001$, ${\eta ^2_p}=0.167$), we conducted the post *post hoc* test for these LDs of each swing phase. The results are shown in Table 3. The LD at maximum foot clearance maximum (Fig. 4e) was significantly narrower on the affected side of the patient with ROA. The LD at toe off minimum (Fig. 4g) was significantly medial in the unaffected lower limb of the patient with ROA. The LD at swing down minimum (Fig. 4i) was significantly more medial on the unaffected side than on the affected side for both patients with LOA and ROA.

#### Temporal gait parameters

The temporal gait parameters of the left and right lower limbs for the HC, patients with LOA and ROA, speed, stride duration, stance duration, and swing duration, are shown in Fig. 3. Mixed ANOVA was performed for the mean of each gait parameter of the HC and patients with LOA and ROA (Supplementary Table S2). Because significant interactions were found in the speed ($F(2,72)=10.74$, $p=<0.001$, ${\eta ^2_p}=0.230$), stance duration ($F(2,72)=25.80$, $p=<0.001$, ${\eta ^2_p}=0.418$), and swing duration($F(2,72)=27.44$, $p=<0.001$, ${\eta ^2_p}=0.433$), we conducted the *post hoc* test for these gait parameters. When the simple effect of the group was found, we conducted a multiple comparison using Shaffer’s method. The results are shown in Table 2. For the stride duration, only the main effect of the group was found in mixed ANOVA ($F(2,72)=16.84$, $p=<0.001$, ${\eta ^2_p}=0.319$); therefore, we conducted the multiple comparison for the groups (Table 2). The speed (Fig. 3c) of the patients with LOA and ROA was significantly slower in both the affected and unaffected sides than that of the HC, but there was no significant difference between the LOA and ROA. The speed was significantly slower in the affected side than in the unaffected side for both the patients with LOA and ROA. The stride duration (Fig. 3d) was significantly longer in the left and right legs of the patients with LOA and ROA than those of the HC, but there was no significant difference in the stride duration between the patients with LOA and ROA. In addition, there was no significant difference between the left and right legs in both the patients with LOA and ROA. The stance duration (Fig. 3e) was significantly longer in both left and right legs of the patients with LOA and ROA than those in the HC, but there was no significant difference between the patients with LOA and ROA. In addition, the stance duration of the patients with LOA and ROA was significantly shorter in the affected side than that in the unaffected one. The swing duration (Fig. 3f) was significantly longer in both left and right legs of the patients with LOA and ROA than that in the HC, but there was no significant difference between the patients with LOA and ROA. The swing duration of the patients with LOA and ROA was significantly longer in the affected side than that in the unaffected side.

**Table 2:** The result of multiple comparison tests for the group.

Gait	Left/	Mean (SD)			p-value			$\eta ^{2}_{p}$
Parameters	Right	HC	LOA	ROA	HCvsLOA	HCvsROA	LOAvsROA	
Maximum foot	Left	0.11 (0.01)	0.11 (0.02)	0.12 (0.01)	No significant simple effect			
Clearance (m)	Right	0.11 (0.02)	0.11 (0.01)	0.11 (0.02)	No significant simple effect			
Stride	Left	1.26 (0.12)	1.06 (0.13)	1.05 (0.11)	< 0.001	< 0.001	0.600	0.389
length (m)	Right	1.26 (0.12)	1.09 (0.14)	1.02 (0.10)	< 0.001	< 0.001	0.048	0.394
Speed (m/s)	Left	1.33 (0.16)	1.01 (0.16)	0.98 (0.16)	< 0.001	< 0.001	0.441	0.513
	Right	1.33(0.16)	1.04(0.17)	0.96(0.15)	< 0.001	< 0.001	0.066	0.512
Stride	Left	0.95 (0.07)	1.07 (0.10)	1.08 (0.10)	No significant main effect			
Duration (s)	Right	0.95 (0.07)	1.07 (0.10)	1.08 (0.10)	No significant main effect			
Stance	Left	0.51 (0.05)	0.56 (0.08)	0.62(0.08)	0.025	< 0.001	0.013	0.245
Duration (s)	Right	0.51 (0.05)	0.60 (0.08)	0.58 (0.07)	< 0.001	< 0.001	0.454	0.239
Swing	Left	0.43 (0.03)	0.50 (0.05)	0.47 (0.03)	< 0.001	0.004	< 0.001	0.385
Duration (s)	Right	0.44 (0.03)	0.47 (0.04)	0.50 (0.05)	0.008	< 0.001	0.007	0.299

For the gait parameters other than the maximum foot clearance and the stride duration, the significant interactions and simple effect of group were found. Thus, the multiple comparison was conducted for the groups for the left and right legs separately. In the maximum foot clearance, a significant interaction was found but the simple effect was no significant. Thus, the result of the multiple comparison for the main effect is shown. Means and (standard deviations) of gait parameters. Healthy control (HC), left hip osteoarthritis (LOA), right hip osteoarthritis (ROA).

**Table 3:** The result of multiple comparison tests for the group.

LDs at swing phase	Left/	Mean (SD)			p-value		
	Right	HC	LOA	ROA	HCvsLOA	HCvsROA	LOAvsROA
LD at	Left	0.03 (0.02)	0.02 (0.01)	0.02 (0.01)	No significant simple effect		
toe off (m)	Right	0.02 (0.02)	0.02 (0.01)	0.02 (0.01)	No significant simple effect		
LD at maximum	Left	0.03 (0.03)	0.03 (0.02)	0.04 (0.02)	No significant simple effect		
foot clearance(m)	Right	0.03 (0.03)	0.03 (0.02)	0.03 (0.02)	No significant simple effect		
LD at	Left	0.01 (0.02)	0.01 (0.01)	0.01 (0.01)	No significant simple effect		
swing down(m)	Right	0.01(0.02)	0.01(0.01)	0.01(0.01)	No significant simple effect		
LD at Toe off	Left	0.06 (0.02)	0.05 (0.03)	0.06 (0.03)	No significant simple effect		
maximum (m)	Right	0.05 (0.02)	0.06 (0.03)	0.05 (0.03)	No significant simple effect		
LD at maximum foot	Left	0.08 (0.04)	0.08 (0.05)	0.09 (0.05)	No significant simple effect		
clearance maximum (m)	Right	0.08 (0.04)	0.09 (0.04)	0.07 (0.04)	No significant simple effect		
LD at swing down	Left	0.04 (0.02)	0.04 (0.02)	0.04 (0.02)	No significant simple effect		
maximum (m)	Right	0.03 (0.02)	0.04 (0.02)	0.03 (0.02)	No significant simple effect		
LD at Toe off	Left	− 0.01 (0.02)	− 0.01 (0.02)	− 0.01(0.03)	No significant simple effect		
minimum (m)	Right	− 0.01 (0.02)	− 0.01 (0.03)	0.00 (0.01)	No significant simple effect		
LD at maximum foot	Left	− 0.03 (0.04)	− 0.01 (0.03)	− 0.02 (0.05)	No significant simple effect		
clearance minimum (m)	Right	− 0.02 (0.04)	− 0.02 (0.04)	− 0.01 (0.03)	No significant simple effect		
LD at swing down	Left	− 0.02 (0.02)	− 0.01 (0.02)	− 0.02 (0.02)	No significant simple effect		
minimum (m)	Right	− 0.01 (0.02)	− 0.02 (0.03)	− 0.01 (0.02)	No significant simple effect		

For the lateral distance (LD) at swing phase other than the toe off maximum, toe off minimum and swing down minimum, significant interactions were found. Means and (standard deviations) of LDs at swing phase. Healthy control (HC), left hip osteoarthritis (LOA), right hip osteoarthritis (ROA).

## Discussion

This study investigated in detail the spatio-temporal parameters in three-dimensional foot trajectory features of female patients with end-stage hip OA using the IMUs attached to the shanks on the affected and unaffected limbs. The results were summarised and the speed of each stride, maximum foot clearance, and the minimum LDs of the foot during swing phase were especially highlighted either for their differences from previous studies or for their novel perspectives.

In the summary of spatial gait parameters, there was no difference in the maximum foot clearance between the patients with hip OA and the HCs while the stride length was significantly shorter in the patients with OA. In addition, in patients with hip OA, the stride length was significantly shorter in the affected side compared to those in the unaffected side. There was no difference in the mean, maximum and minimum LDs of the foot during the swing phase between patients with hip OA and HCs in toe off and maximum foot clearance and swing down. However, the unaffected sides were located more medially than the affected sides at the swing down minimum.

For the temporal gait parameters, the speed of patients with hip OA was significantly slower, and their stride duration, stance duration, and swing duration were significantly longer in the patients with hip OA than those in the HCs. In addition, in patients with hip OA, the speed, and stance duration were significantly decreased on the affected side compared to those on the unaffected side; and the swing duration was significantly longer on affected side than that on the unaffected side. However, there was no significant difference in stride duration between the affected and unaffected sides.

Some of the results basically supported previous studies. The results for stride length, speed, stance duration, and swing duration were consistent with gait characteristics of individuals with hip OA in the previous study^33^. On the other hand, the results for the speed of each stride and the maximum foot clearance differed form those in some previous studies. In measuring the speed of each stride in the left and right lower limbs, there was a significant difference in the stride speed between the affected and unaffected side in the patients with LOA and ROA. This lower speed in the affected side would be caused by a decrease in hip moment^24^ and pain, resulting in a decrease in affected stride length. However, a previous study showed no significant difference in speed between the affected and unaffected sides^34^. This difference between that study and ours is likely caused by the higher radiographic severity of the patients in our study than that in the previous one. Thus, the slower stride speed in the affected side compared with the unaffected side would be associated with heavy radiographic severity. In fact, the hip moment loading asymmetry is associated with OA radiographic severity. Foucher et al. demonstrated that with increasing Kellgren-Lawrence grade, subjects exhibited a greater degree of gait abnormalities known as motion discontinuity^35^.

In the patients with hip OA, the hip extension-flexion angle during gait decreases^36^. From this perspective, we might expect a decrease in the maximum foot clearance. However, the results of this study revealed no significant difference in the maximum foot clearance between the patients with hip OA and HCs, though their stride lengths were shorter than the HCs. Thus, regardless of the small hip extension-flexion angle, the patients with hip OA could raise their foot as high as HCs. In the future, the hip extension-flexion angle and foot trajectory of patients with hip OA during gait should be measured at the same time.

One possible explanation for patients keeping the max foot clearance is as follows. The sagittal view of the pelvis during push-off of hip OA showed 2.5 times more pelvic upward tilt than that of the nonclinical subjects^17^. This large pelvic tilt could guarantee the maximum foot clearance as high as in healthy individuals. In addition, knee joint angle during walking increases in patients with hip OA compared with the HC^25^, which could also guarantee the maximum foot clearance. Another possibility for maintaining the maximum foot clearance could be increased strength of foot muscles. Although the degree of the ankle joint movement was not different in the patients with hip OA compared with that in the HCs^25^, the ankle dorsiflexion increased in both the affected and unaffected sides of the patients with hip OA compared with the HCs. In addition, the electromyogram of the tibialis anterior muscle in the unaffected side also increased in the patients with hip OA^37^. These possibilities should be investigated by measuring more indices relating to gait, including the foot trajectory.

Moreover, the trajectory analysis for the lateral direction importantly suggested a new perspective. LD at the swing down minimum in patients with hip OA was located significantly more medially on the unaffected side than on the affected one. In the frontal plane, there was no significant difference between affected and unaffected sides with respect to hip adduction angle. Thus, the medial LD at swing down minimum would not relate to the ROM of the angle. The medial LD could relate to an exaggerated lateral bending of the pelvic and trunk to affected side during gait in hip OA patients, which is called a Duchenne limp. The Duchenne limp is a movement performed in order to compensate for weakened hip abductor muscles in the affected side to maintain a stance on the affected leg^26,38^. However, this compensation causes the centre of gravity to move to the affected side. Thus, this shift of the centre of gravity is one possible reason for medial LD at swing down minimum in patients with hip OA.

However, the present experiment specifically showed that there was no significant difference in the LD at mean and maximum, but LD at swing down minimum for patients with hip OA was located significantly more medially in the unaffected limb than that in the affected limb. When the unaffected limb swings down in the swing phase, the affected limb is at the terminal stance phase. In patients with hip OA, the anterior femoral head coverage^39^ and peak hip extension angle of the affected limb decrease^26^. Thus, the hip external rotation (femoral external rotation relative to the pelvis) movement could also decrease the anterior coverage of the femoral head during terminal stance, and could be compensated by pelvic rotation, which may lead to a medial trajectory of the swing phase.

The incidence of falls within 1 year is high in female patients with end-stage hip OA, and the main causes of falls are tripping (43.5%) and loss of balance (37.0%)^10^. The causes of tripping have been discussed from the viewpoints of the foot trajectory. For example, the minimum foot clearance (MFC) and minimum toe clearance (MTC) have been reported as risk factors for falls^40,41^. On the contrary, the causes of loss of balance have not been considered within the context of the foot trajectory. The gait features in LD are potentially related to the loss of balance due to the increased sway of the centre of gravity to the left and right. It could be important to instruct the patient to walk with the swing of the unaffected side limb slightly outward to avoid falling. In the future, it is necessary to investigate whether this gait instruction is effective in preventing falls in patients with hip OA and whether this affects the function of the affected side limb.

## Conclusions

This study aimed to investigate the spatio-temporal features, especially, the foot trajectory, in gait parameters of patients with end-stage hip OA using IMUs attached on both shanks. The stride length of the patients with hip OA was significantly shorter on both the affected and unaffected sides than that of the HCs; but maximum foot clearance was not significantly different between the patients with hip OA and HCs. Thus, the foot trajectory of the patient with hip OA was less than the HC only in the forward direction. This result suggests that hip OA compensates the maximum foot clearance in gait as much as healthy people to decrease fall risk. The horizontal and frontal planes foot trajectories are almost symmetrical in HCs, but appear asymmetrical in patients with hip OA. In the horizontal plane foot trajectory of patients with hip OA, the mean LD from the midline of toe off, maximum foot clearance, and swing down during the swing phase were not significantly different from those of HCs, but the swing down minimum of the swing phase in patients with hip OA was located significantly more medially on the unaffected side than on the affected one. The stride length and speed were significantly decreased in both affected and unaffected sides compared with the HC. The stride length and speed were significantly lower in the affected side than in the unaffected side. The stride duration was significantly longer in the hip OA than those of the HC. The stance and swing durations were shorter and longer in the affected side than in the unaffected side. These temporal gait parameters were consistent with gait characteristics of individuals with hip OA in a previous study. In this study, a new gait feature was discovered by detailed evaluation of three-dimensional foot trajectory of female patients end-stage hip OA. In the future, it is necessary to clarify the relationship between these gait characteristics, clinical symptoms, and physical functions.

## Supplementary Information

Supplementary Information.

## Data Availability

The data that support the findings of this study are available from the corresponding author upon reasonable request.
