# Supplementary material for: Three-dimensional foot trajectory in female patients with end-stage hip osteoarthritis during walking
Source: Sci Rep. 2022 Jun 14;12:9843. doi: 10.1038/s41598-022-14070-8 (PMC9198095; doi:10.1038/s41598-022-14070-8)
Supplement: Supplementary file 1 — Supplementary Information. [file 41598_2022_14070_MOESM1_ESM.pdf]

Supplementary information for

Three dimensional foot trajectory in female patients with end-stage hip osteoarthritis during walking

Yu Kiko, Taiki Ogata, Hirotaka Uchitomi, Masaaki Matsubara, Yoshihiro Miyake, and Yoshiaki Wada

Statistical results

The spatial gait parameters

We conducted a mixed ANOVA for the mean of each gait parameter of HC, LOA, and ROA (Supplementary Table S1).

Supplementary Table S1. Spatio-temporal gait parameters mixed ANOVA table.

Gait parameters	Condition	*df*	*F-value*	*p-value*	η_p^2
Maximum foot clearance	Group	2,72	1.02	0.364	0.028
	Left and right	1,72	0.56	0.459	0.008
	Group × left and right	2,72	3.94	0.024	0.099
Stride length	Group	2,72	23.42	<0.001	0.394
	Left and right	1,72	0.09	0.764	0.001
	Group × left and right	2,72	11.82	<0.001	0.247
Speed	Group	2,72	38.23	<0.001	0.515
	Left and right	1,72	0.19	0.666	0.003
	Group × left and right	2,72	10.74	<0.001	0.230
Stride duration	Group	2,72	16.84	<0.001	0.319
	Left and right	1,72	0.69	0.409	0.010
	Group × left and right	2,72	0.78	0.463	0.021
Stance duration	Group	2,72	10.68	<0.001	0.229
	Left and right	1,72	0.02	0.885	<0.001
	Group × left and right	2,72	25.80	<0.001	0.418
Swing duration	Group	2,72	16.78	<0.001	0.318
	Left and right	1,72	<0.01	0.986	<0.001
	Group × left and right	2,72	27.44	<0.001	0.433

The results were as participants: maximum foot clearance: group: $F(2,72) = 1.02$, $p = 0.364$, $\eta_p^2 = 0.028$; left and right: $F(1,72) = 0.56$, $p = 0.459$, $\eta_p^2 = 0.008$; group × left and right interaction: $F(2,72) = 3.94$, $p = 0.024$, $\eta_p^2 = 0.099$. The results of the post hoc test for the group × left and right interaction were as follows: group under the left: $F(2,72) = 0.96$, $p = 0.386$, $\eta_p^2 = 0.026$; group under the right: $F(2,72) = 2.03$, $p = 0.138$, $\eta_p^2 = 0.054$; left and right under the HC: $F(1,24) = 2.63$, $p = 0.118$, $\eta_p^2 = 0.099$; left and right under the LOA: $F(1,24) = 2.53$, $p = 0.125$, $\eta_p^2 = 0.095$; left and right under the ROA: $F(1,24) = 4.00$, $p = 0.057$, $\eta_p^2 = 0.143$.

stride length: group: $F(2,72) = 23.42$, $p < 0.001$, $\eta_p^2 = 0.394$; left and right: $F(1,72) = 0.09$, $p = 0.764$, $\eta_p^2 = 0.001$; group × left and right interaction: $F(2,72) = 11.82$, $p < 0.001$, $\eta_p^2 = 0.247$. The results of the post hoc test for the group × left and right interaction were as follows: group under the left condition: $F(2,72) = 22.88$, $p < 0.001$, $\eta_p^2 = 0.389$; group under the right condition: $F(2,72) = 23.41$, $p < 0.001$, $\eta_p^2 = 0.394$; left and right under the HC condition: $F(1,24) = 0.07$, $p = 0.799$, $\eta_p^2 = 0.003$; left and right under the LOA condition: $F(1,24) = 20.68$, $p < 0.001$, $\eta_p^2 = 0.463$; left and right under the ROA condition: $F(1,24) = 16.14$, $p = 0.001$, $\eta_p^2 = 0.402$. Multiple comparisons between the left and right revealed no significant differences between the LOA and ROA $t(72) = 1.28, p = 0.203$, but revealed significant differences between the HC and LOA conditions, $t(72) = 5.18, p < 0.001$, between the HC and ROA conditions, $t(72) = 6.46, p < 0.001$, under the group. Multiple comparisons between the left and right at the left revealed no significant differences between the LOA and ROA, $t(72) = 0.53, p = 0.600$, but revealed significant differences between the HC and LOA conditions, $t(72) = 5.58, p < 0.001$, between the HC and ROA conditions, $t(72) = 6.10, p < 0.001$, under the group. Multiple comparisons between the left and right at right revealed significant differences between the HC and LOA, $t(72) = 6.80, p < 0.001$, between the HC and ROA, $t(72) = 3.11, p = 0.003$ and between the LOA and ROA, $t(72) = 3.68, p < 0.001$, under the group.

The temporal gait parameters

A mixed ANOVA was performed for the mean, maximum, and minimum of each gait parameter for HC, LOA, and ROA (Supplementary Table S1 and S2)

The results were as participants: speed: group: $F(2,72) = 38.23$, $p < 0.001$, $\eta_p^2 = 0.515$; left and right: $F(1,72) = 0.19$, $p = 0.666$, $\eta_p^2 = 0.003$; group × left and right interaction: $F(2,72) = 10.74$, $p < 0.001$, $\eta_p^2 = 0.230$. The results of the post hoc test for the group × left and right interaction were as follows: group under the left: $F(2,72) = 38.00$, $p < 0.001$, $\eta_p^2 = 0.513$; group under the right: $F(2,72) = 37.69$, $p < 0.001$, $\eta_p^2 = 0.512$; left and right under the HC: $F(1,24) = 0.05$, $p = 0.833$, $\eta_p^2 = 0.002$; left and right under the LOA: $F(1,24) = 19.27$, $p < 0.001$, $\eta_p^2 = 0.445$; left and right under the ROA: $F(1,24) = 15.96$, $p = 0.001$, $\eta_p^2 = 0.399$. Multiple comparisons between the left and right revealed no significant differences between the LOA and ROA $t(72) = 1.33, p = 0.187$, but significant differences between the HC and LOA, $t(72) = 6.82, p < 0.001$, between the HC and ROA conditions, $t(72) = 8.15, p < 0.001$, under the group. Multiple comparisons between the left and right at the left revealed no significant differences between the LOA and ROA, $t(72) = 0.77, p = 0.441$, but revealed significant differences between the HC and LOA, $t(72) = 7.13, p < 0.001$, between the HC and ROA, $t(72) = 7.90, p < 0.001$, under the group. Multiple comparisons between the left and right at the right revealed no significant differences between the LOA and ROA, $t(72) = 1.87, p = 0.066$, but revealed significant differences between the HC and LOA, $t(72) = 6.41, p < 0.001$, between the HC and ROA, $t(72) = 8.28, p < 0.001$, under the group. stride duration: group: $F(2,72) = 16.84$, $p < 0.001$, $\eta_p^2 = 0.319$; left and right: $F(1,72) = 0.69$, $p = 0.409$, $\eta_p^2 = 0.010$; group × left and right interaction: $F(2,72) = 0.78$, $p = 0.463$, $\eta_p^2 = 0.021$. Multiple comparisons between the left and right revealed no significant differences between the LOA and ROA $t(72) = 0.57, p = 0.567$, but revealed significant differences between the HC and LOA conditions, $t(72) = 4.71, p < 0.001$, between the HC and ROA, $t(72) = 5.29, p < 0.001$, under the group.

stance duration: group: $F(2,72) = 10.68$, $p < 0.001$, $\eta_p^2 = 0.229$; left and right: $F(1,72) = 0.02$, $p = 0.885$, $\eta_p^2 < 0.001$; group × left and right interaction: $F(2,72) = 25.80$, $p < 0.001$, $\eta_p^2 = 0.418$. The results of the post hoc test for the group × left and right interaction were as follows: group under the left: $F(2,72) = 11.70$, $p < 0.001$, $\eta_p^2 = 0.245$; group under the right: $F(2,72) = 11.28$, $p < 0.001$, $\eta_p^2 = 0.239$; left and right under the HC: $F(1,24) = 1.39$, $p = 0.250$, $\eta_p^2 = 0.055$; left and right under the LOA: $F(1,24) = 17.24$, $p < 0.001$, $\eta_p^2 = 0.418$; left and right under the ROA: $F(1,24) = 22.67$, $p < 0.001$, $\eta_p^2 = 0.486$. Multiple comparisons between the left and right revealed no significant differences between the LOA and ROA $t(72) = 0.96, p = 0.340$, but revealed significant differences between the HC and LOA , $t(72) = 3.43, p = 0.001$, between the HC and ROA conditions,$t(72) = 4.39, p < 0.001$, under the group. Multiple comparisons between the left and right at the left showed significant differences between the HC and LOA, $t(72) = 2.29, p = 0.025$, between the HC and ROA, $t(72) = 4.84, p < 0.001$, between the LOA and ROA, $t(72) = 2.54, p = 0.013$, under the group. Multiple comparisons between the left and right at the right revealed no significant differences between the LOA and ROA, $t(72) = 0.75, p = 0.454$, but revealed significant differences between the HC and LOA, $t(72) = 4.44, p < 0.001$, between the HC and ROA, $t(72) = 3.69, p < 0.001$, under the group. swing duration: group: $F(2,72) = 16.78$, $p < 0.001$, $\eta_p^2 = 0.318$; left and right: $F(1,72) < 0.01$, $p = 0.986$, $\eta_p^2 < 0.001$; group × left and right interaction: $F(2,72) = 27.44$, $p < 0.001$, $\eta_p^2 = 0.433$. The results of the post hoc test for the group × left and right interaction were as follows: group under the left: $F(2,72) = 22.50.$, $p < 0.001$, $\eta_p^2 = 0.385$; group under the right: $F(2,72) = 15.34$, $p < 0.001$, $\eta_p^2 = 0.299$; left and right under the HC: $F(1,24) = 1.41$, $p = 0.247$, $\eta_p^2 = 0.055$; left and right under the LOA: $F(1,24) = 18.34$, $p < 0.001$, $\eta_p^2 = 0.433$; left and right under the ROA: $F(1,24) = 23.85$, $p < 0.001$, $\eta_p^2 = 0.498$. Multiple comparisons between the left and right revealed no significant differences between the LOA and ROA $t(72) = 0.48, p = 0.633$, but revealed significant differences between the HC and LOA, $t(72) = 5.24, p < 0.001$, between the HC and ROA conditions, $t(72) = 4.75, p < 0.001$, under the group. Multiple comparisons between the left and right at left showed significant differences between the HC and LOA, $t(72) = 6.69, p < 0.001$, between the HC and ROA, $t(72) = 2.98, p = 0.004$, between the LOA and ROA, $t(72) = 3.71, p < 0.001$, under the group. Multiple comparisons between the left and right at right showed significant differences between the HC and LOA, $t(72) = 2.73, p = 0.008$, between the HC and ROA, $t(72) = 5.54, p < 0.001$, between the LOA and ROA, $t(72) = 2.80, p = 0.007$, under the group.

The results were as participants: LD at toe off: group: $F(2,72) = 0.79$, $p = 0.458$, $\eta_p^2 = 0.022$; left and right: $F(1,72) = 0.67$, $p = 0.416$, $\eta_p^2 = 0.009$; group × left and right interaction: $F(2,72) = 0.42$, $p = 0.660$, $\eta_p^2 = 0.012$.

LD at maximum foot clearance: group: $F(2,72) = 0.24$, $p = 0.783$, $\eta_p^2 = 0.007$; left and right: $F(1,72) = 1.31$, $p = 0.257$, $\eta_p^2 = 0.018$; group × left and right interaction: $F(2,72) = 0.25$, $p = 0.778$, $\eta_p^2 = 0.007$.

LD at swing down: group: $F(2,72) = 0.15$, $p = 0.857$, $\eta_p^2 = 0.004$; left and right: $F(1,72) = 0.05$, $p = 0.820$, $\eta_p^2 = 0.001$; group × left and right interaction: $F(2,72) = 0.73$, $p = 0.483$, $\eta_p^2 = 0.020$.

LD at toe off maximum: group: $F(2,72) = 0.05$, $p = 0.948$, $\eta_p^2 = 0.002$; left and right: $F(1,72) = 1.02$, $p = 0.315$, $\eta_p^2 = 0.014$; group × left and right interaction: $F(2,72) = 3.70$, $p = 0.029$, $\eta_p^2 = 0.093$. The results of the post hoc test for the group × left and right interaction were as follows: group under the left: $F(2,72) = 1.60$, $p = 0.210$, $\eta_p^2 = 0.042$; group under the right: $F(2,72) = 0.89$, $p = 0.416$, $\eta_p^2 = 0.024$; left and right under the HC: $F(1,24) = 1.45$, $p = 0.240$, $\eta_p^2 = 0.057$; left and right under the LOA: $F(1,24) = 1.90$, $p = 0.180$, $\eta_p^2 = 0.074$; left and right under the ROA: $F(1,24) = 4.21$, $p = 0.051$,

Supplementary Table S2. Lateral distance (LD) of each swing phase mixed ANOVA table.

LDs at swing phase	Condition	*df*	*F-value*	*p-value*	η_p^2
LD at toe off	Group	2,72	0.79	0.458	0.022
	Left and right	1,72	0.67	0.416	0.009
	Group × left and right	2,72	0.42	0.660	0.012
LD at maximum foot clearance	Group	2,72	0.24	0.783	0.007
	Left and right	1,72	1.31	0.257	0.018
	Group × left and right	2,72	0.25	0.778	0.007
LD at swing down	Group	2,72	0.15	0.857	0.004
	Left and right	1,72	0.05	0.820	0.001
	Group × left and right	2,72	0.73	0.483	0.020
LD at toe off maximum	Group	2,72	0.05	0.948	0.002
	Left and right	1,72	1.02	0.315	0.014
	Group × left and right	2,72	3.70	0.029	0.093
LD at maximum foot clearance maximum	Group	2,72	0.17	0.840	0.005
	Left and right	1,72	1.37	0.245	0.019
	Group × left and right	2,72	2.77	0.070	0.071
LD at swing down maximum	Group	2,72	0.35	0.706	0.010
	Left and right	1,72	0.06	0.810	0.001
	Group × left and right	2,72	2.49	0.090	0.065
LD at toe off minimum	Group	2,72	0.16	0.852	0.004
	Left and right	1,72	0.76	0.386	0.011
	Group × left and right	2,72	3.59	0.033	0.091
LD at maximum foot clearance minimum	Group	2,72	0.43	0.651	0.012
	Left and right	1,72	0.35	0.558	0.005
	Group × left and right	2,72	1.95	0.149	0.052
LD at swing down minimum	Group	2,72	0.18	0.834	0.005
	Left and right	1,72	0.35	0.558	0.005
	Group × left and right	2,72	7.20	0.001	0.167

$\eta_p^2 = 0.149$.

LD at maximum foot clearance maximum: group: $F(2,72) = 0.17$, $p = 0.840$, $\eta_p^2 = 0.005$; left and right: $F(1,72) = 1.37$, $p = 0.245$, $\eta_p^2 = 0.019$; group × left and right interaction: $F(2,72) = 2.77$, $p = 0.070$, $\eta_p^2 = 0.071$. The results of the post hoc test for the group × left and right interaction were as follows: group under the left: $F(2,72) = 0.54$, $p = 0.583$, $\eta_p^2 = 0.015$; group under the right: $F(2,72) = 1.75$, $p = 0.181$, $\eta_p^2 = 0.046$; left and right under the HC: $F(1,24) = 0.76$, $p = 0.393$, $\eta_p^2 = 0.031$; left and right under the LOA: $F(1,24) = 0.82$, $p = 0.374$, $\eta_p^2 = 0.033$; left and right under the ROA: $F(1,24) = 4.62$, $p = 0.041$, $\eta_p^2 = 0.162$.

LD at swing down maximum: group: $F(2,72) = 0.35$, $p = 0.706$, $\eta_p^2 = 0.010$; left and right: $F(1,72) = 0.06$, $p = 0.810$, $\eta_p^2 = 0.001$; group × left and right interaction: $F(2,72) = 2.49$, $p = 0.090$, $\eta_p^2 = 0.065$.

LD at toe off minimum: group: $F(2,72) = 0.16$, $p = 0.852$, $\eta_p^2 = 0.004$; left and right: $F(1,72) = 0.76$, $p = 0.386$, $\eta_p^2 = 0.011$; group × left and right interaction: $F(2,72) = 3.59$, $p = 0.0330$, $\eta_p^2 = 0.091$. The results of the post hoc test for the group × left and right interaction were as follows: group under the left: $F(2,72) = 0.85$, $p = 0.431$, $\eta_p^2 = 0.023$; group under the right: $F(2,72) = 2.75$, $p = 0.071$, $\eta_p^2 = 0.071$; left and right under the HC: $F(1,24) = 0.55$, $p = 0.467$, $\eta_p^2 = 0.022$; left and right under the LOA: $F(1,24) = 2.21$, $p = 0.150$, $\eta_p^2 = 0.084$; left and right under the ROA: $F(1,24) = 5.38$, $p = 0.029$, $\eta_p^2 = 0.183$.

LD at maximum foot clearance minimum: group: $F(2,72) = 0.43$, $p = 0.651$, $\eta_p^2 = 0.012$; left and right: $F(1,72) = 0.35$, $p = 0.558$, $\eta_p^2 = 0.005$; group × left and right interaction: $F(2,72) = 1.95$, $p = 0.149$, $\eta_p^2 = 0.052$.

LD at swing down minimum: group: $F(2,72) = 0.18$, $p = 0.834$, $\eta_p^2 = 0.005$; left and right: $F(1,72) = 0.35$, $p = 0.558$, $\eta_p^2 = 0.005$; group × left and right interaction: $F(2,72) = 7.20$, $p = 0.001$, $\eta_p^2 = 0.167$. The results of the post hoc test for the group × left and right interaction were as follows: group under the left: $F(2,72) = 2.18$, $p = 0.121$, $\eta_p^2 = 0.057$; group under the right: $F(2,72) = 2.92$, $p = 0.061$, $\eta_p^2 = 0.075$; left and right under the HC: $F(1,24) = 0.92$, $p = 0.347$, $\eta_p^2 = 0.037$; left

Supplementary Table S3. Comparison of physical function between affected and unaffected limbs of patients with hip OA. MJS and JOA hip score depicts the minimum joint space and Japanese Orthopedic Association hip score respectively.

	Median (Interquartile range)		*p-value*
	Affected limb (N = 50)	Unaffected limb (N = 50)	
Hip flexion (degree)	87.5 (80-95)	105 (100-113.8)	<0.001
Hip extension (degree)	10 (5-10)	10 (10-15)	<0.001
Hip abduction (degree)	15 (10-20)	27.5 (20-33.8)	<0.001
Hip adduction (degree)	10 (5-15)	15 (10-20)	<0.001
Hip external rotation (degree)	20 (15-28.8)	30 (20-40)	<0.001
Hip internal rotation (degree)	20 (5-30)	35 (30-43.8)	<0.001
Hip abductor strength (kgf/kg)	0.16 (0.13-0.21)	0.20 (0.16-0.25)	<0.001
Knee extension muscle strength (kgf/kg)	0.27 (0.22-0.34)	0.33 (0.28-0.38)	<0.001
MJS (mm)	0 (0-0.5)	3.6 (2.4-4.0)	<0.001
JOA hip score (total 100points)	65 (57-75)	91 (86-94)	<0.001

and right under the LOA: $F(1,24) = 7.15$, $p = 0.013$, $\eta_p^2 = 0.223$; left and right under the ROA: $F(1,24) = 7.45$, $p = 0.012$, $\eta_p^2 = 0.237$.

Supplementary Table S4. The median and interquartile range of physical functions of affected and unaffected limbs and comparison of affected/unaffected limbs between the patients with LOA and ROA. The minimal joint space (MJS) were determined by the orthopedic surgeon using simple radiograph. The Japanese Orthopedic Association hip score (JOA hip score). * indicates a significant difference of 5%.

	Median (Interquartile range)				*p-value*	
	Affected limb		Unaffected limb			
	LOA	ROA	LOA	ROA	Affected vs Affected	Unaffected vs Unaffected
Hip flexion (degree)	90<br>(80-100)	85<br>(80-90)	105<br>(100-110)	110<br>(100-115)	0.388	0.806
Hip extension (degree)	10<br>(5-10)	10<br>(5-10)	10<br>(10-15)	10<br>(10-15)	0.814	0.772
Hip abduction (degree)	15<br>(10-20)	20<br>(10-25)	30<br>(15-30)	25<br>(20-35)	0.307	0.238
Hip adduction (degree)	15<br>(10-15)	10<br>(5-15)	15<br>(10-20)	10<br>(10-15)	0.028*	0.218
Hip external rotation (degree)	20<br>(15-25)	20<br>(15-30)	30<br>(20-35)	30<br>(20-45)	0.235	0.690
Hip internal rotation (degree)	20<br>(5-25)	20<br>(5-30)	35<br>(30-45)	35<br>(25-40)	0.665	0.206
Hip abductor strength (kgf/kg)	0.17<br>(0.14-0.21)	0.15<br>(0.13-0.18)	0.20<br>(0.17-0.25)	0.21<br>(0.14-0.25)	0.375	0.660
Knee extension muscle strength (kgf/kg)	0.27<br>(0.22-0.34)	0.27<br>(0.22-0.31)	0.33<br>(0.26-0.41)	0.33<br>(0.29-0.36)	0.956	0.782
MJS (mm)	0<br>(0-0.4)	0<br>(0-0.5)	3.0<br>(2.0-4.3)	3.8<br>(3.0-4.0)	0.535	0.446
JOA hip score (total 100 points)	65<br>(57-75)	65<br>(57-76)	90<br>(86-94)	91<br>(86-94)	0.854	0.690
Hip pain of gait (mm)	29<br>(0-54)	29<br>(7-54)			0.785	

Supplementary Table S5. Contingency table for the stage of hip OA for affected and affected sides of the patients with LOA and ROA. The p-values for the affected and unaffected sides between the patients with LOA and ROA were 0.883 and 0.584, respectively.

Stage of hip OA	Affected limb		Unaffected limb	
	LOA	ROA	LOA	ROA
Normal	0	0	1	0
Pre-	1	0	9	12
Early-	2	2	9	10
Advanced-stage	3	5	4	3
Severe	19	18	2	0
